# Identification of a Sudden Cardiac Death Susceptibility Locus at 2q24.2 through Genome-Wide Association in European Ancestry Individuals

**DOI:** 10.1371/journal.pgen.1002158

**Published:** 2011-06-30

**Authors:** Dan E. Arking, M. Juhani Junttila, Philippe Goyette, Adriana Huertas-Vazquez, Mark Eijgelsheim, Marieke T. Blom, Christopher Newton-Cheh, Kyndaron Reinier, Carmen Teodorescu, Audrey Uy-Evanado, Naima Carter-Monroe, Kari S. Kaikkonen, Marja-Leena Kortelainen, Gabrielle Boucher, Caroline Lagacé, Anna Moes, XiaoQing Zhao, Frank Kolodgie, Fernando Rivadeneira, Albert Hofman, Jacqueline C. M. Witteman, André G. Uitterlinden, Roos F. Marsman, Raha Pazoki, Abdennasser Bardai, Rudolph W. Koster, Abbas Dehghan, Shih-Jen Hwang, Pallav Bhatnagar, Wendy Post, Gina Hilton, Ronald J. Prineas, Man Li, Anna Köttgen, Georg Ehret, Eric Boerwinkle, Josef Coresh, W. H. Linda Kao, Bruce M. Psaty, Gordon F. Tomaselli, Nona Sotoodehnia, David S. Siscovick, Greg L. Burke, Eduardo Marbán, Peter M. Spooner, L. Adrienne Cupples, Jonathan Jui, Karen Gunson, Y. Antero Kesäniemi, Arthur A. M. Wilde, Jean-Claude Tardif, Christopher J. O'Donnell, Connie R. Bezzina, Renu Virmani, Bruno H. C. h. Stricker, Hanno L. Tan, Christine M. Albert, Aravinda Chakravarti, John D. Rioux, Heikki V. Huikuri, Sumeet S. Chugh

**Affiliations:** 1 McKusick-Nathans Institute of Genetic Medicine, Johns Hopkins University School of Medicine, Baltimore, Maryland, United States of America; 2 Institute of Clinical Medicine, Department of Internal Medicine, University of Oulu, Oulu, Finland; 3 Division of Cardiology, Miller School of Medicine, University of Miami, Miami, Florida, United States of America; 4 Montreal Heart Institute and the Université de Montréal, Montreal, Canada; 5 The Heart Institute, Cedars-Sinai Medical Center, Los Angeles, California, United States of America; 6 Department of Epidemiology, Erasmus MC, Rotterdam, The Netherlands; 7 Heart Failure Research Center, Department of Clinical and Experimental Cardiology, Academic Medical Center, University of Amsterdam, Amsterdam, The Netherlands; 8 Center for Human Genetic Research, Massachusetts General Hospital, Boston, Massachusetts, United States of America; 9 Cardiology Division, Massachusetts General Hospital, Boston, Massachusetts, United States of America; 10 Framingham Heart Study, National Heart, Lung, and Blood Institute, National Institutes of Health, Framingham, Massachusetts, United States of America; 11 CVPath Institute, Gaithersburg, Maryland, United States of America; 12 Department of Internal Medicine, Erasmus MC, Rotterdam, The Netherlands; 13 Netherlands Consortium for Healthy Aging (NCHA), Netherlands Genomic Initiative (NGI), Rotterdam, The Netherlands; 14 Division of Cardiology, Department of Medicine, Johns Hopkins University School of Medicine, Baltimore, Maryland, United States of America; 15 Division of Public Health Sciences, Wake Forest University School of Medicine, Winston-Salem, North Carolina, United States of America; 16 Department of Epidemiology, Johns Hopkins University, Baltimore, Maryland, United States of America; 17 Cardiology, Department of Medicine, Geneva University Hospital, Geneva, Switzerland; 18 University of Texas Health Science Center at Houston, Houston, Texas, United States of America; 19 Departments of Medicine and Biostatistics, Johns Hopkins University, Baltimore, Maryland, United States of America; 20 Cardiovascular Health Research Unit, Departments of Medicine and Epidemiology, University of Washington, Seattle, Washington, United States of America; 21 Group Health Research Institute, Group Health Cooperative, Seattle, Washington, United States of America; 22 Cardiovascular Health Research Unit, Division of Cardiology, Department of Medicine, University of Washington, Seattle, Washington, United States of America; 23 Department of Biostatistics, Boston University School of Public Health, Boston, Massachusetts, United States of America; 24 Department of Emergency Medicine, Oregon Health and Science University, Portland, Oregon, United States of America; 25 Department of Pathology, Oregon Health and Science University, Portland, Oregon, United States of America; 26 Biocenter Oulu, University of Oulu, Oulu, Finland; 27 National Heart, Lung, and Blood Institute, Bethesda, Maryland, United States of America; 28 Department of Medical Informatics, Erasmus MC, Rotterdam, The Netherlands; 29 Inspectorate of Health Care, The Hague, The Netherlands; 30 Center for Arrhythmia Prevention, Division of Preventive Medicine, Cardiovascular Division, Department of Medicine, Brigham and Women's Hospital, Harvard Medical School, Boston, Massachusetts, United States of America; University of Oxford, United Kingdom

## Abstract

Sudden cardiac death (SCD) continues to be one of the leading causes of mortality worldwide, with an annual incidence estimated at 250,000–300,000 in the United States and with the vast majority occurring in the setting of coronary disease. We performed a genome-wide association meta-analysis in 1,283 SCD cases and >20,000 control individuals of European ancestry from 5 studies, with follow-up genotyping in up to 3,119 SCD cases and 11,146 controls from 11 European ancestry studies, and identify the *BAZ2B* locus as associated with SCD (*P* = 1.8×10^−10^). The risk allele, while ancestral, has a frequency of ∼1.4%, suggesting strong negative selection and increases risk for SCD by 1.92–fold per allele (95% CI 1.57–2.34). We also tested the role of 49 SNPs previously implicated in modulating electrocardiographic traits (QRS, QT, and RR intervals). Consistent with epidemiological studies showing increased risk of SCD with prolonged QRS/QT intervals, the interval-prolonging alleles are in aggregate associated with increased risk for SCD (*P* = 0.006).

## Author Summary

Family studies have clearly demonstrated a role for genes in modifying risk for sudden cardiac death (SCD), however genetic studies have been limited by available samples. Here we have assembled over 4,400 SCD cases with >30,000 controls, all of European ancestry, and utilize a two-stage study design. In the first stage, we conducted an unbiased genome-wide scan in 1,283 SCD cases and >20,000 controls, and then performed follow-up genotyping in the remainder of the samples. We demonstrate strong association to a region of the genome not previously implicated in SCD, the *BAZ2B* locus, which contains 3 genes not previously known to play a role in cardiac biology. In addition, we used the genome-wide scan data to test a focused hypothesis that genetic variants that modulate ECG traits associated with SCD (QT, QRS, and RR intervals) also modify risk for SCD, and we demonstrate that QT- and QRS-prolonging alleles are, as a group, associated with increased risk of SCD. Taken together, these findings begin to elucidate the genetic contribution to SCD susceptibility and provide important targets for functional studies to investigate the etiology and pathogenesis of SCD.

## Introduction

Despite recent progress in treatment and prevention of coronary heart disease, sudden cardiac death (SCD) remains a major public health problem, with an annual incidence of SCD that ranges from 50 to 100 per 100,000 in the general population [1], [2]. While there has been a great deal of focus on SCD in the setting of Mendelian forms of arrhythmia (e.g. long and short QT syndromes), the vast majority of SCD events occur in the general population, with up to 50% of individuals manifesting SCD as a first sign of disease [3]. An estimated ∼80% of all SCDs are associated with coronary disease, ∼10–15% in the setting of cardiomyopathy and ∼5% occur in persons with myocarditis, coronary anomalies or ion channelopathies (e.g. long QT/Brugada/short QT syndromes) [4]. Despite this clinical heterogeneity, a familial component to SCD risk has been demonstrated even after adjusting for traditional cardiovascular disease risk factors [5], [6], [7], [8], suggesting that genetic factors are likely to play an important role. The critical importance of the genetic contribution for effective prediction and prevention of SCD has been emphasized in a recent consensus document from the US National Heart Lung and Blood Institute [9].

In this study, we perform a meta-analysis of 5 genome-wide association studies (GWAS) with follow-up genotyping in up to 11 additional studies of European ancestry to identify genetic variants that modify susceptibility to community-based SCD. In addition to an unbiased scan of the genome, we also focus on specific SNPs that have been previously associated with electrophysiological traits that, when extreme, are associated with increased risk for SCD (QRS, QT, and RR intervals).

## Results

To identify genetic determinants of SCD, genome-wide genotyping and imputation of ∼2.5 million SNPs was performed in 1,283 SCD cases and ∼20,000 controls drawn from 5 samples of European ancestry: Atherosclerosis Risk in Communities (ARIC), Framingham Heart Study (FHS), FinGesture, Oregon Sudden Unexpected Death Study (Oregon-SUDS), Rotterdam Study (RS) (Table S1). All individual studies showed minimal test statistic inflation after post-imputation quality control (genomic control λ<1.03) (see Materials and Methods). Meta-analysis was performed using inverse variance weighting, with minimal test statistic inflation observed (λ = 1.004) and no early departure from the null expectation (Figure S1A), suggesting that overall there was good genotyping quality and minimal population substructure. Two loci exceeded the genome-wide significance threshold of *P*<5×10^−8^ (Figure S1B, Table 1).

**Table 1:** Summary of GWAS and follow-up genotyping results for association with SCD.

SNP	Chr	Position	Coded/Non-coded allele	AF	GWAS OR (95% CI)	GWAS P	Follow-up OR (95% CI)	Follow-up P	Combined P
**rs174230**	**2**	**159,883,556**	**T/C**	**0.013**	**2.49 (1.78–3.47)**	**8.58E-08**	**1.38 (0.99–1.93)**	**0.03**	3.0E-07
**rs4665058**	**2**	**159,898,455**	**A/C**	**0.014**	**2.52 (1.80–3.53)**	**7.07E-08**	**1.48 (1.05–2.08)**	**0.01**	5.8E-08
rs4665058*							**1.65 (1.29–2.12)**	**8.0E-05**	**1.8E-10**
rs16880395	4	27,848,761	T/C	0.230	1.30 (1.16–1.46)	5.14E-06	0.97 (0.87–1.07)	0.27	0.01
rs2178490	5	30,875,088	G/A	0.207	1.32 (1.17–1.48)	5.63E-06	0.90 (0.81–1.00)	NA	0.11
rs12517578	5	106,008,730	G/C	0.234	0.76 (0.67–0.85)	4.41E-06	0.94 (0.85–1.04)	0.12	0.0001
rs3193970	10	97,061,998	C/T	0.421	0.78 (0.71–0.86)	1.11E-06	0.96 (0.89–1.05)	0.2	0.0001
rs11626637	14	45,792,412	G/A	0.100	0.64 (0.52–0.78)	9.57E-06	1.00 (0.87–1.16)	NA	0.01
rs2650907	16	75,950,708	G/C	0.421	0.75 (0.68–0.83)	4.39E-08	1.03 (0.92–1.13)	NA	0.0005
rs7218928	17	32,338,069	G/A	0.429	0.79 (0.72–0.87)	4.34E-06	1.02 (0.94–1.11)	NA	0.008
rs12601622	17	73,560,970	A/G	0.014	6.79 (3.43–13.42)	3.69E-08	0.89 (0.64–1.23)	NA	0.08
rs6507566	18	39,939,723	T/G	0.330	1.26 (1.14–1.40)	7.09E-06	0.93 (0.85–1.02)	NA	0.08

Chr, chromosome; AF, allele frequency of coded allele (weighted by study size); OR, odds ratio; CI, confidence interval. Follow-up genotyping results are reported for 1,730 SCD cases and 10,530 controls, with the exception of rs12601622 (1,460 SCD cases, 10.182 controls), which failed genotyping in the Oregon-SUDS follow-up study. Bold indicates nominal significance (*P*<0.05) for validation. *P*-values for validation are reported as one-sided, and NA indicates opposite direction of effect from GWAS. *Includes ARREST (719 SCD cases, 4,190 controls) and AGNES (670 SCD cases, 654 controls) studies.

There were 13 independent loci containing at least 1 SNP with *P*<10^−5^ (Table S2). We attempted to follow-up all of these loci in additional independent case-control samples from FinGesture and Oregon-SUDS, as well as in seven additional cohorts: Cardiovascular Health Study (CHS), CVPath Institute Sudden Cardiac Death registry (CVPI-SCDr), the Harvard Cohorts (5 combined cohorts, see Materials and Methods). In total, this follow-up genotyping included 1,730 cases and 10,530 controls of European ancestry (Table S3). We were able to design assays and obtain high quality genotype data for 11 SNPs corresponding to 10 of these independent loci (Table 1). Nominally significant replication results consistent with the GWAS were observed for two highly correlated SNPs (r^2^ = 1 in HapMap CEU samples) in the *BAZ2B* (bromodomain adjacent zinc finger domain 2B) locus (rs174230, *P* = 0.03; rs4665058, *P* = 0.01). On the other hand there was no significant evidence of replication for the two loci that were of genome-wide significance in the initial scan (rs2650907 and rs12601622 located on chromosomes 16 and 17, respectively), suggesting that they represent either false positive signals in the initial scan or insufficient power of the replication cohort. In this context, it is important to note that the imputation quality of rs12601622 was poor across all studies (imputation quality (r^2^)<0.4).

**Figure 1:**
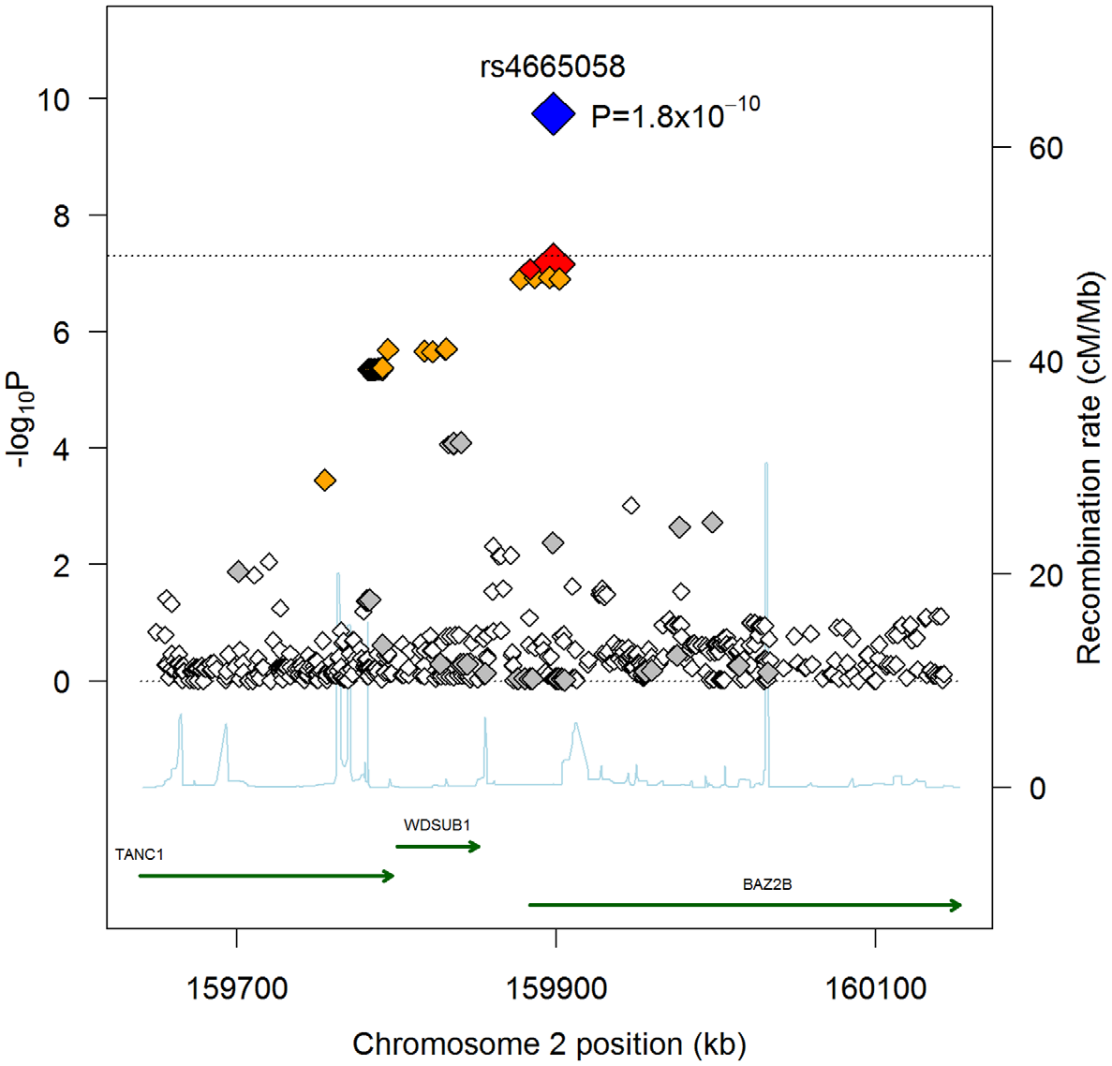
Regional association plot for rs4655058. Each SNP is plotted with respect to its chromosomal location (x-axis) and *P*-value (y-axis on the left). The tall blue spikes indicate the recombination rate (y-axis on the right) at that region of the chromosome. The index SNP is denoted by the larger diamond, for both the GWAS (red) and combined GWAS and validation results (blue). The dotted black line denotes genome-wide significance (*P*<5×10^−8^). Shading of additional SNPs indicates degree of linkage disequilibrium with the index SNP (red: r^2^≥0.8, orange: 0.5≤r^2^<0.8, grey: 0.2≤r^2^<0.5, white: r^2^<0.2).

When combining follow-up results with the discovery GWAS results, only rs4665058 approached genome-wide significance (*P* = 5.8×10^−8^). This marker was therefore further genotyped in the ARREST study, which includes 719 SCD cases, and in 4,190 population-based controls, and in the recently published AGNES case-control study [10], which is a study of 670 individuals with first MI and ventricular fibrillation (VF) compared to 654 with MI alone. In the combined follow-up cohorts, including the ARREST and AGNES studies, this SNP was significant after Bonferroni correction for the number of loci tested (*P_nominal_* = 8.0×10^−5^; *P_corrected_* = 8.0×10^−4^). In a combined analysis of GWAS and all follow-up genotyping results, rs4665058 well exceeded genome-wide significance (*P* = 1.8×10^−10^) (Figure 1). The risk allele (A allele) of rs4665058 has a study size weighted frequency of 1.4%, and increases risk for SCD by 1.92-fold per allele (95% CI 1.57 to 2.34) in the combined analysis (Figure S2), and by 1.65-fold per allele (95% CI 1.29 to 2.12) in the follow-up samples alone (Table 1). No significant heterogeneity was observed (Q statistic  = 15.6, *P* = 0.11; I^2^ = 35.9, 95% CI 0 to 68.5%). No significant interaction was observed for either sex or age (data not shown). It is interesting to note that the risk allele is the ancestral allele (based on non-human primate sequence), and its low frequency in European ancestry populations suggests strong negative selection, as fewer than 0.8% of ancestral alleles have reached a frequency of 1.4% or lower (Figure S3).

In addition to performing an unbiased GWAS in the 5 discovery cohorts, we also used the results from the initial GWAS meta-analysis to examine the role of SNPs previously reported to be associated with QRS (ventricular depolarization), QT (ventricular depolarization/repolarization), and/or RR (inverse heart rate) intervals [11], [12], [13], [14]. These electrocardiographic measured traits are associated with cardiovascular mortality and SCD in both Mendelian settings (e.g. LQTS), and in the general population [15], [16], [17]. Overall, there were 49 independent loci associated with the ECG traits reported (25 for QRS [14], 16 QT [12], [13], 9 for RR [11], with one locus overlapping between QRS and QT intervals) (Tables S4, S5, S6). While in general, we hypothesize that QRS/QT/heart rate prolonging alleles increase risk for SCD, recent pleiotropy analyses of ECG traits have shown inconsistent directions of effects for some alleles (e.g. a QT-prolonging allele is associated with decreased QRS interval) [14], and thus, *a priori* we have chosen a two-sided test to assess significance of association for ECG SNPs with SCD. Nominal significance was observed for 3 loci, including *PLN* (QT/QRS, *P* = 0.013), *NOS1AP* (QT, *P* = 0.010), and *KCNQ1* (QT, *P* = 0.014). A fourth locus, *TKT/CACNA1D/PRKCD* (QRS, *P* = 0.0007), was significant after multiple-test correction for all 49 tested SNPs (corrected *P* = 0.034) (Table 2). Interestingly, the direction of effect for *TKT/CACNA1D/PRKCD* and *KCNQ1* is opposite of that expected: the QRS/QT interval prolonging allele is associated with decreased risk for SCD. For the remaining 2 loci, the direction of effect is consistent with a model in which increasing QRS/QT interval increases risk for SCD. Indeed, looking across all 49 loci (Tables S4, S5, S6), we find that in aggregate QRS/QT/heart rate(inverse RR)-prolonging alleles are more often associated with increased risk for SCD (31/49; *P* = 0.03). This result is entirely driven by QRS (17/25, *P* = 0.04) (Table S4) and QT intervals (12/16, *P* = 0.02) (Table S5), with no overrepresentation observed for RR interval (3/9, *P* = NS) (Table S6).

**Table 2:** Association of QRS/QT interval associated SNPs with SCD.

Trait	Nearest Gene	Index SNP	Chr	Position	Coded/Non-coded Allele	Trait β	SCD OR (95% CI)	SCD P	Concordant Effect
**QRS**	**TKT/CACNA1D/PRKCD**	**rs4687718**	**3**	**53,257,343**	**A/G**	**−0.63**	**1.27 (1.10–1.45)**	**0.0007**	**NO**
QRS	C6orf204/SLC35F1/PLN/BRD7P3	^§^rs11153730	6	118,774,215	C/T	0.59	1.13 (1.02–1.25)	0.013	YES
QT	NOS1AP	rs12143842	1	160,300,514	T/C	2.88	1.16 (1.03–1.3)	0.010	YES
QT	PLN	^§^rs11970286	6	118,787,067	T/C	1.64	1.11 (1.01–1.22)	0.037	YES
QT	KCNQ1	rs12296050	11	2,445,918	T/C	1.44	0.85 (0.76–0.96)	0.014	NO

Chr, chromosome; OR, odds ratio; CI, confidence interval. Results are drawn from the SCD GWAS only (n = 1283 case, >20,000 controls). Trait beta estimates (β) are in milliseconds (ms). P-values are for a two-tailed test. Concordant Effect refers to whether the QRS/QT prolonging allele is associated with increased risk of SCD. Bold indicates significant after Bonferroni correction for the 49 SNPs tested. QRS results are drawn from [14] and QT results are drawn from the QTSCD study [13]. **§** These SNP represent the same genetic effect (r^2^ = 0.91).

The results for QRS and QT intervals (combined *P* = 0.006) are consistent with epidemiological studies, which show increased QRS/QT interval in the general population is associated with increased risk of SCD. While the general trend is significant, the observation that 2/4 of the individual SNPs nominally associated with SCD actually show the opposite direction of effect, including *TKT/CACNA1D/PRKCD*, suggests the need to further elucidate how each of these variants modifies both the underlying QRS/QT interval trait as well as the potential role in SCD. For example, it is possible that the risk for SCD is not directly (or entirely) mediated though the effect on QRS/QT interval, as has been suggested for *NOS1AP* [18].

## Discussion

Using meta-analysis of GWAS with follow-up genotyping in independent samples, we demonstrate strong evidence for SCD susceptibility at locus 2q24.2 in individuals of European ancestry. Somewhat surprisingly, we identified a relatively rare allele (MAF = 0.014) with a strong effect (OR = 1.92), which is in contrast to most GWAS for complex traits, which have typically identified alleles with MAF >0.1 and ORs<1.5. Given concerns about imputation accuracy for rarer alleles, we note that rs4665058 is almost perfectly in LD with rs174230 (r^2^ = 1.0 in CEU), which is directly genotyped in all the GWAS samples, and shows almost identical association results (Table 1). Further, we observe the same effect size for FinGesture GWAS and follow-up genotyping samples (Figure S2), again suggesting that imputation does not affect the results. Indeed, it is quite plausible that rare variants play a large role in risk for SCD given the fatal nature of SCD (∼10% of SCD victims survive) potentially selecting against more common risk alleles.

Despite the strong evidence for association with SCD across the entire study, we note that no effect for rs4665058 is observed in the AGNES and CHS studies. Power was 63% and 52%, respectively, under an additive genetic model with OR = 1.92, requiring a nominal P = 0.05. We observe no significant heterogeneity in the meta-analysis including all studies (P = 0.11), suggesting that the lack of association in these two cohorts is likely a function of random chance, magnified by the low frequency of the risk allele. We would also point out that AGNES has a more narrow phenotype than the other studies, and included only those who suffered their first MI and survived the initial event to receive in-hospital care, which could contribute to the lack of association. For CHS, SCD adjudication was performed along with the ARIC samples, and thus represents an identical phenotype. The only difference was the age of the individuals in these two cohorts (ARIC 45–64 years, CHS >65), however in interaction analyses we did not observe age to be a significant modifier of the genetic effect.

As with other GWAS, the current study design is not without limitations. First, association approaches depend upon linkage disequilibrium to identify associated SNPs, thus the underlying functional variant at any of the loci is potentially unrecognized. Indeed, using Pilot 1 data from the 1000 Genomes project (November 2010 release) [19], we do not identify any missense mutations highly correlated with the most strongly associated SNP, rs4665058 (r^2^>0.8), suggesting that the functional variant is likely to be regulatory. To test whether rs4665058 was an eQTL, we searched the GTEx eQTL database (http://www.ncbi.nlm.nih.gov/gtex/test/GTEX2/gtex.cgi), which queries lymphoblastoid, liver and various brain regions. We did not observe any eQTLs for rs4665058 or any SNPs from the 1000 Genomes data highly correlated with rs4665058 (r^2^>0.8). We recognize that this negative finding is not necessarily informative, given that expression from heart tissue has not been queried. Second, we only implicate loci, as opposed to genes, and additional work is required to definitively identify which gene at a locus is responsible. Indeed, while the strongest association signal maps to an intron in *BAZ2B*, due to linkage disequilibrium, the signal also extends to the *WDSUB1* and *TANC1* genes. All three genes are expressed in human heart (http://www.genecards.org) and the mouse heart during various key stages during cardiogenesis and the formation of the autonomic nervous system from neural crest (Embryonic day, E7.5–10.5) (http://biogps.gnf.org). *BAZ2B* and *WDSUB1* are essentially uncharacterized, however, *TANC1* has been shown to regulate dendritic spines and excitatory synapses in both cultured neurons and a mouse knock-out [20]. *TANC1* is most highly expressed in heart in humans, however, no cardiac phenotype in the TANC1 knock-out mouse was noted. Third, while we present evidence that in aggregate QRS/QT interval-prolonging alleles are associated with SCD, only *TKT/CACNA1D/PRKCD* exceeds a Bonferroni corrected P-value threshold, suggesting inadequate power for these analyses. The findings related to individual QRS/QT interval-prolonging alleles should therefore be considered exploratory and require replication in additional populations. Finally, we note that the meta-analysis consisted of both population-based and case-control studies, with some of the case-control studies using CAD controls as opposed to population-based controls (Tables S1 and S3). The inclusion of different control groups allows us to separate out whether the risk conferred by the *BAZ2B* locus acts through increased risk for CAD, which is present in ∼80% of SCD victims. The consistent results in studies with CAD controls (FinGesture GWAS, Oregon-SUDS GWAS, Oregon-SUDS replication) (Figure S2) provide evidence that the risk associated with rs4665058 may be specific to SCD, rather than a generic risk factor for CAD. Indeed, given the case mix across the cohorts (primary ventricular fibrillation, ischemic CAD, non-ischemic CAD, non-CAD), the study is best powered to identify variants that increase risk of SCD through a mechanism common across the various subtypes of SCD.

In summary, we have identified the locus including the bromodomain-containing gene [21], *BAZ2B,* as a new SCD susceptibility locus. The bromodomain is an exclusive protein domain known to recognize acetyl-lysine residues on proteins and might play an important role in chromatin remodeling and gene transcription regulation [22]. The risk allele, while low frequency in Caucasian populations (MAF  = 0.014) has a relatively large effect, increasing risk for SCD by >1.9-fold per allele (95% CI 1.57 to 2.34). While rs4665058 may not be clinically relevant in the general population in whom the increment in absolute risk attributable to the variant is modest, exploring its role in high-risk populations (e.g. heart failure, SQTS/LQTS) may help to identify those who could benefit from intervention. Beyond the *BAZ2B* locus, our study also highlights the role of QRS/QT interval associated variants in the risk of SCD, and suggests that larger GWAS of these and other intermediate risk factors may yield additional SCD loci.

## Materials and Methods

### Participating Studies

Five studies consisting of individuals of European ancestry from Europe and the United States contributed to the GWAS discovery phase of this study: Atherosclerosis Risk in Communities (ARIC), Framingham Heart Study (FHS), FinGesture, Oregon-Sudden Unexpected Death Study (Oregon-SUDS), and Rotterdam Study (RS). For follow-up, additional genotyping was performed in independent samples from FinGesture and Oregon-SUDS, as well as in 8 additional populations of European ancestry: AmsteRdam REsuscitation STudies (ARREST), Cardiovascular Health Study (CHS), CVPath Institute Sudden Cardiac Death registry (CVPI-SCDr), and Harvard Cohorts (consisting of 5 combined populations, see below). We also performed a look-up of our top result in the AGNES study. All studies received approval from the appropriate institutional review committees, and the subjects in each cohort provided written informed consent.

### Discovery Studies

#### ARIC

The ARIC study includes 15,792 men and women from four communities in the United States (Jackson, Mississippi; Forsyth County, North Carolina; Washington County, Maryland; suburbs of Minneapolis, Minnesota) enrolled in 1987–1989 and prospectively followed [23]. Assessment of SCD has been previously described [18]. Briefly, all cases of fatal CHD that occurred by December 31, 2002 were reviewed and adjudicated by a committee of physicians. SCD was operationally defined as a sudden pulseless condition from a cardiac origin in a previously stable individual, and the reviewers classified each CHD death as definite sudden arrhythmic death, possible sudden arrhythmic death, definite non-sudden death, or unclassifiable. The primary outcome of SCD described in the present study combines both definite and possible sudden arrhythmic death. For the present analysis, participants were censored at time of loss to follow up or death if the cause of death was other than SCD.

#### FHS

The Framingham Heart Study is a longitudinal cohort study including individuals recruited from three generations recruited without regard to phenotype and followed up with serial clinical examinations, mailed updates and review of medical records. The SCD adjudication was conducted by three physicians using previously established criteria. A SCD was defined as a coronary heart disease death within one hour of the onset of symptoms.

#### FinGesture

The FinGesture study started in 1999 aimed at collecting consecutive victims of out-of-hospital sudden death from a defined geographical area, Oulu University Hospital District in northern Finland. All the victims of sudden death were autopsied at the Department of Forensic Medicine, University of Oulu, Oulu, Finland. The definition of SCD caused by an acute coronary event has been previously described in detail [8]. In each case of sudden death, the mechanism of death was defined and all patients who were considered to have died due to any cause other than SCD due to an acute coronary event were excluded from the study. Of the out-of-hospital SCD victims, those with (1) a witnessed sudden death within 6 hours of the onset of the symptoms or within 24 hours of the time that the victim was last seen alive in a normal state of health and (2) evidence of a coronary complication, defined as a fresh intracoronary thrombus, plaque rupture or erosion, intraplaque hemorrhage, or critical coronary stenosis (>75%) in the main coronary artery were included in the SCD group. Victims of SCD with other serious heart diseases, such as severe valve disease or cardiomyopathy, were excluded. In addition, victims with evidence of non-cardiac causes and victims with mechanical causes of sudden death, such as a rupture of the myocardium and/or tamponade, extensive myocardial necrosis (>50%), rupture of entire papillary muscle, pulmonary edema, or any cause of death considered to be due to some reason other than ischemia-induced SCD, were also excluded.

For the discovery GWAS, the FinGesture study control population consisted of MI patients from the same geographical area than cases and treated in the University of Oulu Hospital [24]. Acute myocardial infarction was diagnosed according to ICD-10 classification with at least two of three following findings: elevated troponin/ckMbm levels, typical angina pectoris, EKG ST-segment changes typical for MI. All patients who had in-hospital life-threatening ventricular arrhythmias were excluded from the study. The replication control group consisted of subjects without a history of coronary heart disease, AMI, or aborted cardiac arrest from the OPERA (Oulu Project Elucidating Risk of Atherosclerosis) study [25]. These general population samples were randomly selected subjects from the social insurance register covering the entire population of the city of Oulu, Finland. The mean age of the subjects at the beginning of the study was 51 years.

#### Oregon-SUDS

The Oregon Sudden Unexpected Death Study (ongoing since 2002), is a community-based study of SCD among residents of the Portland, Oregon metropolitan area (pop. approx. 1,000,000). Methods of case ascertainment have been published earlier [1],[26]. In brief, patients with SCD were ascertained from the regional emergency medical response system (EMS), the County Medical Examiner, and emergency departments of the 16 area hospitals. Determination of SCD was made after in-house adjudication of all cases based on the arrest circumstances detailed in the EMS incident report or medical examiner report (available for all cases), medical records (available for 79% of cases) and autopsy data (available for 15% of cases). SCD was defined as a sudden unexpected pulse-less condition of likely cardiac origin and survivors of SCA were included. If un-witnessed, SCD subjects were included if they were found dead within 24 hours of having last been seen alive and in normal state of health. Subjects were excluded if they had a chronic terminal illness (e.g. terminal cancer), or an identifiable non-cardiac etiology of sudden death related to trauma, overdose, drowning or suicide. Cases included in the current GWAS study were white non-Hispanic SCA cases with DNA available (a blood or tissue sample was available in 59% of cases). Case subjects were also required to have documented significant coronary artery disease (CAD), or, if aged ≥50 years, were assumed to have CAD (based on 95% likelihood of CAD in SCA cases aged ≥50 years) [27]. Significant CAD was defined as ≥50% stenosis of a major coronary artery from an angiogram prior to arrest or at autopsy; physician report of past MI; history of percutaneous coronary intervention (PCI) or coronary artery bypass grafting (CABG); autopsy-identified CAD; or MI by clinical data with any two of the following three: ischemic symptoms, positive troponins or CKMB; or pathologic Q waves on ECG. For the discovery GWAS, controls were drawn from the ARIC cohort, and consisted of 1,208 individuals with prevalent or non-fatal incident CAD. The ARIC and Oregon-SUDS genotype data was combined before imputation, and QC for both individuals and SNPs performed on the combined samples. Oregon-SUDS controls analyzed in the replication samples are subjects from the same geographic region who had coronary artery disease but no history of SCD (n = 348).

#### Rotterdam Study

The Rotterdam Study is an ongoing prospective population-based cohort study of chronic diseases in Caucasian elderly, which started in 1990. The Medical Ethics Committee of the Erasmus University approved the study. All inhabitants of Ommoord, a Rotterdam suburb in the Netherlands, aged 55 years and over (n = 10,278) were invited to participate. Of them, 78% (n = 7,983) gave their written informed consent for participation. Baseline examinations took place from March 1990 through July 1993. All participants were continuously monitored for major morbidity and mortality through linkage with general practitioner and municipality records. Detailed information on design, objectives and methods of the Rotterdam Study is described elsewhere [28]. Of all 7,983 participants, 5,974 subjects were genotyped on the Infinium II HumanHap550K Genotyping BeadChip® version 3 (Illumina) as part of a large population-based project on genetics of complex traits and diseases. The ascertainment of SCD cases in the Rotterdam Study has been described previously [17]. SCD cases were defined as a witnessed natural death attributable to cardiac causes, heralded by abrupt loss of consciousness, within one hour of onset of acute symptoms, or as an unwitnessed, unexpected death of a person seen in a stable medical condition within 24 hours before death without evidence of a non-cardiac cause.

### Follow-Up Studies

#### AGNES

The AGNES case-control set consists of individuals with a first acute ST-elevation myocardial infarction [5]. AGNES cases had ECG-registered ventricular fibrillation occurring before reperfusion therapy for an acute and first ST-elevation myocardial infarction. AGNES controls were individuals with a first acute ST-elevation myocardial infarction but without ventricular fibrillation. All cases and controls were recruited at seven heart centers in The Netherlands from 2001–2010. We excluded individuals with an actual non–ST-elevation myocardial infarction, prior myocardial infarction, congenital heart defects, known structural heart disease, severe comorbidity, electrolyte disturbances, trauma at presentation, recent surgery, previous coronary artery bypass graft or use of class I and III antiarrhythmic drugs. Individuals who developed ventricular fibrillation during or after percutaneous coronary intervention were not eligible. Furthermore, because early reperfusion limits the opportunity of developing ventricular fibrillation, potential control subjects undergoing percutaneous coronary intervention within 2 h after onset of myocardial ischemia symptoms were not included. This time interval was based on the observation that >90% of cases developed ventricular fibrillation within 2 h after onset of the complaint of symptoms.

#### ARREST

ARREST is an ongoing prospective population-based study that covers >95% of all out-of-hospital cardiac arrests (with ECG documentation) in a contiguous region of the Netherlands with ∼2.4 million inhabitants, that was designed to study the clinical and genetic determinants of sudden cardiac death [10], [29]. In collaboration with all Emergency Medical Services (EMS) in this study region, all patients with out-of-hospital cardiac arrest (OHCA) with ECG-documented ventricular tachycardia/fibrillation (VT/VF) are prospectively included. To ensure >95% coverage, a data collection infrastructure has been set up that records all CPR attempts with EMS involvement for OHCA from ambulance dispatch to discharge from the hospital or to death according to the Utstein template. This method presumably reflects the real-life situation better than studies that only include SCD victims who survive and are admitted to the hospital. SCD was defined as OHCA due to cardiac causes with ECG-documentation of VT/VF. Medical history and current disease diagnosis are retrieved from the patient's General Practitioner and/or hospital records, and medication use prior to the resuscitation is retrieved from the patient's pharmacist. For the current study, 719 OHCA cases with VT/VF were included. Controls were drawn from the RS-II and RS-III cohorts from the Rotterdam Study [28].

#### CVPI-SCDr

The CVPath Institute Sudden Cardiac Death registry samples are received through and ongoing joint consultation service provided to the Maryland Office of the Chief Medical Examiner initiated in 1993. Sudden death is defined as symptoms commencing within 6 hours of death (witnessed arrest) or death occurring within 24-hours after the victim was last seen alive in his normal state of health. Comprehensive analysis of each sample includes coronary artery histology, and cases of unexpected sudden death are stratified into cardiac deaths (with coronary disease (CAD): at least 1 epicardial coronary artery has ≥75% cross-sectional luminal narrowing by an atherosclerotic plaque or a lesion with a superimposed thrombus or evidence of a prior MI and no other cause of death; non-CAD: atherosclerosis with less <75% cross-sectional luminal narrowing (non-flow limiting CAD) and/or cardiomyopathies) and non-cardiac deaths (e.g., drug overdose, trauma, seizure disorder, stroke). All samples are genotyped for a panel of ancestry informative markers, and only those identified as Caucasian by the CVPI-SCDR with concordant genotype data are included. For the current study, 259 sudden cardiac deaths were included, and all non-SCD CHS samples were used as population-based controls.

#### CHS

CHS is a population-based prospective cohort study of cardiovascular disease, and includes 5,888 participants >65 years of age identified from four U.S. communities using Medicare eligibility lists. The original cohort included 5201 participants recruited in 1989–1990 and 687 additional subjects were recruited in 1992–1993 to enhance the racial/ethnic diversity of the cohort [30]. The following exclusion criteria were applied to obtain the final sample for the present analysis: no consent for genetic analyses, poor quality DNA (samples with < 60% of genotypes called), and self-described ethnicity other than White. Assessment of SCD was identical to that of ARIC, described above, with all cases of fatal CHD that occurred by July 31, 2002 examined.

#### Harvard Cohorts

The study design is a case-control investigation sampled from prospective cohorts and clinical trials, taking advantage of the time-to-event data by matching cases and controls on follow-up time. The SCD cases from the Physicians' Health Study (PHS I and II), the Nurses' Health Study (NHS), the Health Professionals Follow-up Study (HPFS), and the Women's Antioxidant Cardiovascular Study (WACS) were included in the present analysis. In all cohorts, cases of sudden and/or arrhythmic cardiac death are confirmed by medical record review (hospital, emergency room, autopsy, and emergency medical services reports) and next-of-kin descriptions of the circumstances surrounding the death. The definition of SCD has been previously described [31]. Briefly, a cardiac death is considered a definite SCD if the death or cardiac arrest that precipitated death occurred within one hour of symptom onset as documented by medical records or next-of-kin reports or had an autopsy consistent with SCD (i.e. acute coronary thrombosis or severe coronary artery disease without myocardial necrosis or other pathologic findings to explain death). Deaths were also classified as arrhythmic based on the definition of Hinkle and Thaler [32]. Unwitnessed deaths or deaths that occurred during sleep were considered probable SCDs if the participant was documented to be symptom free when last observed within the preceding 24 hours, and circumstances suggested that the death could have been sudden. A total of 435 confirmed sudden and arrhythmic cardiac deaths among individuals of self-described white ancestry were included in the analysis. Controls were selected using risk-set sampling [33], with up to three controls for each case matched on study cohort, sex, age (+/−1 year), ethnicity, smoking status (current, never, past), time and date of blood sampling, fasting status, and presence or absence of cardiovascular disease (MI, angina, CABG, or stroke) prior to death.

### Phenotype Modeling

For all studies, covariates, measured at baseline for prospective cohorts, included age and gender, with the following exceptions: FinGesture GWAS included the top 10 principal components calculated from pre-imputation genotype data through a multi-dimensional scaling (MDS) method, as implemented in PLINK [34]; CVPI-SCDr did not include covariates, as the controls were population-based controls from CHS.

### Genome-Wide Genotyping and Imputation

Genotyping was performed using either Affymetrix or Illumina arrays, depending on the cohort (Table S7). Each study performed filtering of both individuals and SNPs to ensure robustness for genetic analysis. SNP genotypes were assessed for quality, and SNPs failing quality control were removed before imputation according to specific criteria (Table S7). Each study utilized the remaining SNP genotypes to impute genotypes for approximately 2.5 million autosomal SNPs based on linkage disequilibrium patterns observed in the HapMap CEU samples (Utah residents of Northern and Western European descent). Imputed genotypes were calculated as dosages, with fractional values between 0 and 2 reflecting the estimated number of copies of a given allele for a given SNP for each individual. The use of dosages allows for the incorporation of the uncertainty in the imputations into subsequent analysis. All studies used a hidden Markov model as implemented in the MACH software [35]. All results are reported on the forward strand. Post-imputation, quantile-quantile (QQ) plots were generated for each study, stratified by both allele frequency and imputation quality to identify classes of SNPs that show strong early departure from the null. Based on these analyses, for the ARIC and Rotterdam studies, SNPs with minor allele frequency (MAF) <0.01 were excluded. For FHS, SNPs with MAF <0.02 and/or imputation quality score <0.40 were excluded.

### Follow-Up Genotyping

Genotyping was performed using iPlex single base primer extension with MALDI-TOF mass spectrometry according to manufacturer protocols (Sequenom Inc., San Diego, CA). SNPs were excluded from each study if call rate was ≤90% or Hardy-Weinberg equilibrium *P*<0.001. SNPs were excluded from final meta-analysis if fewer than 4 out of 5 studies reported back results for that SNP. PCR and extension primer sequences are available upon request. In ARREST, rs4665058 was genotyped using TaqMan (Applied Biosystems), and primer and probe sequences are available upon request. In AGNES, genotypes at rs4665058 were determined by direct genotyping (N = 360) using Taqman assay or imputation from HapMap reference panel (N = 969) using the Markov-chain Monte Carlo method implemented in MACH1.0 (Rsq = 0.97). Details on genotype imputation have been described elsewhere [10].

### Statistical Methods

For prospective community-based samples, associations between SCD and SNPs were tested using Cox proportional hazards regression models under the assumption of an additive model of genotypic effect. For case-control samples, a logistic regression framework was employed. For the Harvard cohorts, risk set analysis was used to match cases and controls holding time at risk stable; conditional logistic regression in risk set sampling avoids differences in time at risk that otherwise result. These models were adjusted for age and sex. In family-based cohorts (FHS), linear mixed modeling was implemented to additionally control for relatedness [36]. A genomic control correction factor (λ), calculated from all imputed SNPs, was applied on a per-study basis to account for cryptic population sub-structure and other potential biases [37]. Regression results were meta-analyzed using inverse variance weighted fixed-effects models as implemented in the METAL software package (http://www.sph.umich.edu/csg/abecasis/metal/). Results were considered statistically significant at a *P*-value of 5×10^−8^, a figure that reflects the estimated testing burden of one million independent SNPs in samples of European ancestry [38]. For age by SNP and sex by SNP interactions, we performed regression analysis as described above separately within each study, including both main effects and an interaction term in the model. Meta-analysis of discovery and replication results was performed using inverse-variance weighting as implemented in the R package ‘meta’ (R version 2.81, http://www.r-project.org/). To account for the age range differences across the cohorts, we used a random-effects model for the age by SNP interaction.

## Supporting Information

Figure S1 Association results for GWAS for SCD in 5 populations of European ancestry. (A) The QQ plot shows no early departure from the null expectation between observed and expected P-values; (B) Manhattan plot. Dotted line indicates threshold for genome-wide significance (P<5×10^−8^).
(TIF)

Figure S2 Forest plot for rs4665058. Note that FHS does not report data for this SNP. Freq incidates the coding allele frequency, TE indicates the beta estimate, and seTE is the standard error of the beta estimate.
(TIF)

Figure S3 Ancestral allele (based on comparison to non-human primate sequence) frequency distribution in HapMap CEU. Red line indicates allele frequency of the rs4665058 risk allele (A allele).
(TIFF)

Table S1 GWAS cohort chracteristics. Age for prospective studies is at baseline.
(PDF)

Table S2 Summary of GWAS and validation results for association with SCD. Chr, chromosome; AF, allele frequency of coded allele (study size weighted average). Follow-up genotyping results are reported for 1,730 SCD cases and 10,530 controls, with the exception of rs12601622 (1,460 SCD cases, 10.182 controls), which failed genotyping in the Oregon-SUDS follow-up study. Bold indicates nominal significance (*P*<0.05) for validation. P-values for validation are reported as one-sided, and NA indicates opposite direction of effect from GWAS. *Includes ARREST (719 SCD cases, 4,190 controls) and AGNES (670 SCD cases, 654 controls) studies. Follow-up genotyping results are reported for the 11 SNPs which passed genotyping QC.
(PDF)

Table S3 Follow-up genotyping cohort characteristics. Age for prospective studies is at baseline.
(PDF)

Table S4 Association of QRS interval associated SNPs with SCD. Chr, chromosome; OR, odds ratio; CI, confidence interval. Trait beta estimates (β) are in milliseconds (ms). P-values are for a two-tailed test. Bold indicates nominal significance (P<0.05). Concordant Effect refers to whether the QRS prolonging allele is associated with increased risk of SCD. QRS interval results are drawn from Sootodehnia et al.^14^. ^§^This SNPs represent the same genetic effect for QT as rs11970286 in Table S5 (r^2^ = 0.91).
(PDF)

Table S5 Association of QT interval associated SNPs with SCD. Chr, chromosome; OR, odds ratio; CI, confidence interval. Trait beta estimates (β) are in milliseconds (ms). P-values are for a two-tailed test. Bold indicates nominal significance (*P*<0.05). Concordant Effect refers to whether the QT prolonging allele is associated with increased risk of SCD. QT results are drawn from the QTSCD study^13^, unless otherwise noted. *Genome-wide significant results (*P*<5×10^-8^) are drawn from the QTGEN study^12^, and standardized beta estimates and SE were converted to ms using SD = 17.5 ms. ^§^This SNP represent the same genetic effect for QRS interval as rs11153730 in Table S4 (r^2^ = 0.91).
(PDF)

Table S6 Association of RR interval associated SNPs with SCD. Chr, chromosome; OR, odds ratio; CI, confidence interval. Trait beta estimates (β) are in milliseconds (ms). P-values are for a two-tailed test. Concordant Effect refers to whether the QT prolonging allele is associated with increased risk of SCD. RR results are drawn from^11^. ^§^This SNP is partially correlated with rs11153730 and rs11970286 from Tables S4 and S5, respectively (r^2^ = 0.59).
(PDF)

Table S7 Study genome-wide genotyping characteristics.
(PDF)
