# Supplementary figures and images for: Identification of a Sudden Cardiac Death Susceptibility Locus at 2q24.2 through Genome-Wide Association in European Ancestry Individuals
Source: PLoS Genet. 2011 Jun 30;7(6):e1002158. doi: 10.1371/journal.pgen.1002158 (PMC3128111; doi:10.1371/journal.pgen.1002158)

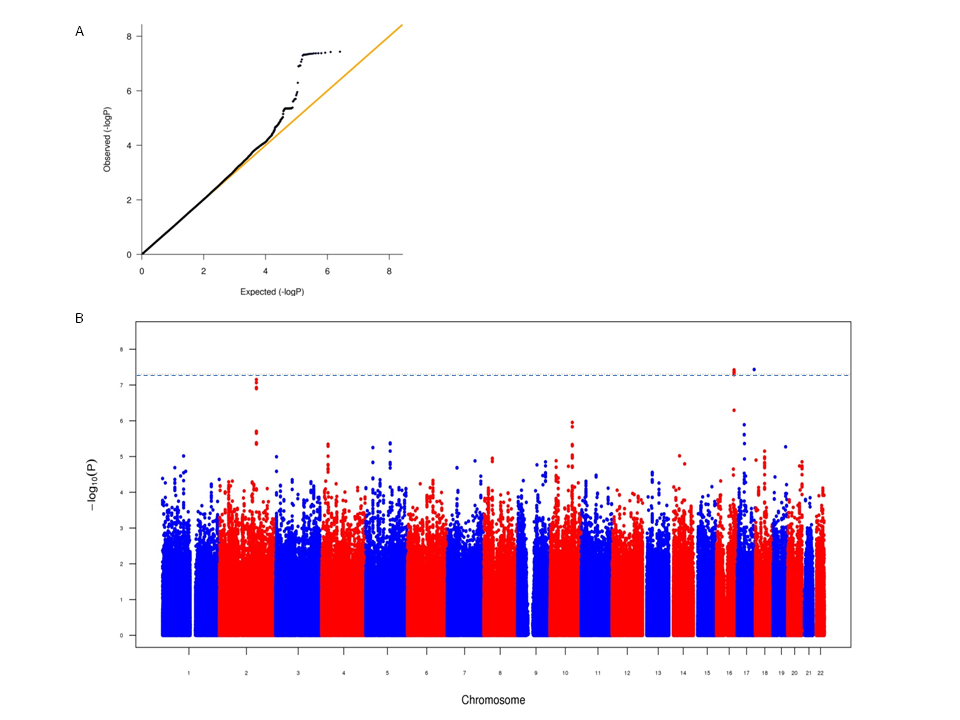

Supplement: Figure S1 — Association results for GWAS for SCD in 5 populations of European ancestry. (A) The QQ plot shows no early departure from the null expectation between observed and expected P-values; (B) Manhattan plot. Dotted line indicates threshold for genome-wide significance (P<5×10^−8^). (TIF) [file pgen.1002158.s001.tif]

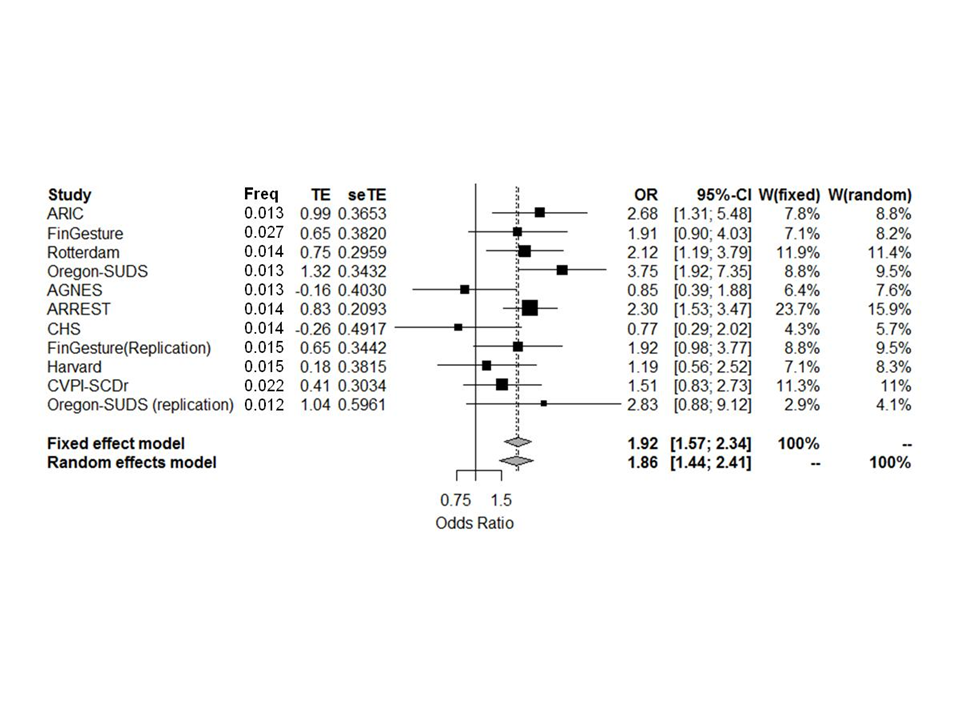

Supplement: Figure S2 — Forest plot for rs4665058. Note that FHS does not report data for this SNP. Freq incidates the coding allele frequency, TE indicates the beta estimate, and seTE is the standard error of the beta estimate. (TIF) [file pgen.1002158.s002.tif]

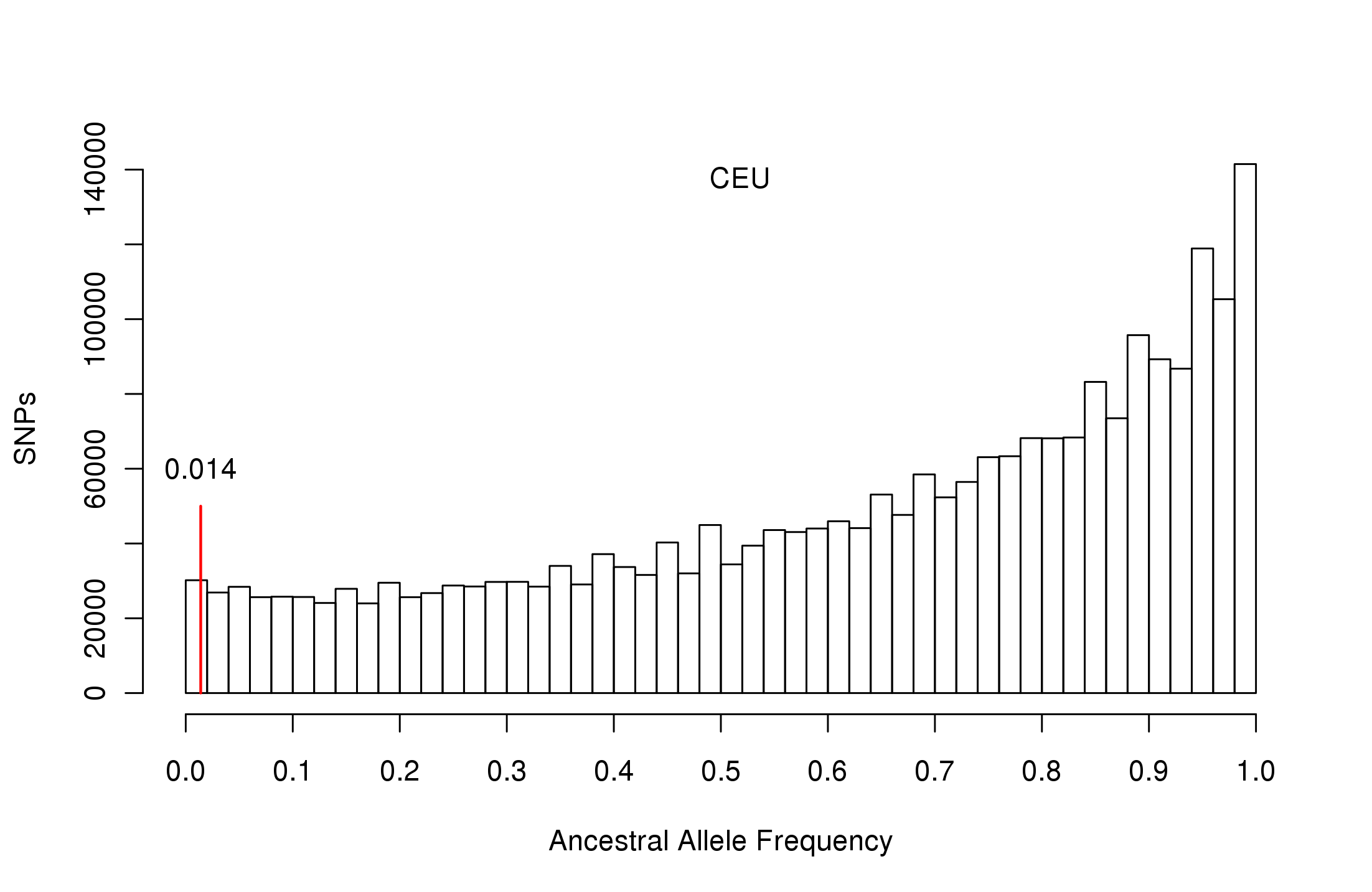

Supplement: Figure S3 — Ancestral allele (based on comparison to non-human primate sequence) frequency distribution in HapMap CEU. Red line indicates allele frequency of the rs4665058 risk allele (A allele). (TIFF) [file pgen.1002158.s003.tif]
