# Supplementary material for: Identification of a Sudden Cardiac Death Susceptibility Locus at 2q24.2 through Genome-Wide Association in European Ancestry Individuals
Source: PLoS Genet. 2011 Jun 30;7(6):e1002158. doi: 10.1371/journal.pgen.1002158 (PMC3128111; doi:10.1371/journal.pgen.1002158)
Supplement: Table S1 — GWAS cohort chracteristics. Age for prospective studies is at baseline. (PDF) [file pgen.1002158.s004.pdf]

**Supplementary Table 1**. GWAS cohort chracteristics.

| Characteristic | ARIC | FHS | FinGesture | Rotterdam | Oregon-SUDS |
|---|---|---|---|---|---|
| N, Participants with genotype data | 9747 | 4390 | 1039* | 5974 | 1775** |
| N, Participants after exclusion | 9006 | 4390 | 910 | 5974 | 1775 |
| Sex, women, % | 53.0 | 54.99 | 20.5 | 59.4 | 28.5 |
| Age, years, mean | 54.3 | 64.96 | 62.2 | 69.4 | 68.2 |
| Age, years, range | 44 – 66 | 30 – 100 | 28-92 | 55-99 | 36 – 81 |
| N, Sudden cardiac death | 124 | 32 | 340 | 220 | 567 |
| Average time to SCD (years) | 7.82 | 3.78 | NA | 7.27 | NA |
| Mean follow up time (years) | 16.24 | 5.56 | NA | 10.98 | NA |
| Prospective/Case-control | Prospective | Prospective | Case-control | Prospective | Case-control |
| SCD Definition/Ascertainment | sudden pulseless condition from a cardiac origin in a previously stable individual, review of death and medical records | coronary heart disease death within one hour of onset of symptoms, review of death and medical records | out-of-hospital sudden death with evidence of a coronary complication, witnessed within 6 hours of symptoms or seen alive in normal state within 24 hours, autopsy-determined | Witnessed natural death attributable to cardiac causes, within 1 hour of symptoms, or within 24 hours of normal state if unwitnessed, medical and death records | Sudden unexpected pulseless condition of likely cardiac origin, if unwitnessed, seen alive and in normal state of health within 24 hours, collected through EMS, review of death and medical records |
| Control Definition | NA | NA | MI survivors | NA | ARIC individuals with non-fatal CAD |

Age for prospective studies is at baseline.
