# Supplementary material for: Identification of a Sudden Cardiac Death Susceptibility Locus at 2q24.2 through Genome-Wide Association in European Ancestry Individuals
Source: PLoS Genet. 2011 Jun 30;7(6):e1002158. doi: 10.1371/journal.pgen.1002158 (PMC3128111; doi:10.1371/journal.pgen.1002158)
Supplement: Table S2 — Summary of GWAS and validation results for association with SCD. Chr, chromosome; AF, allele frequency of coded allele (study size weighted average). Follow-up genotyping results are reported for 1,730 SCD cases and 10,530 controls, with the exception of rs12601622 (1,460 SCD cases, 10.182 controls), which failed genotyping in the Oregon-SUDS follow-up study. Bold indicates nominal significance (P<0.05) for validation. P-values for validation are reported as one-sided, and NA indicates opposite direction of effect from GWAS. *Includes ARREST (719 SCD cases, 4,190 controls) and AGNES (670 SCD cases, 654 controls) studies. Follow-up genotyping results are reported for the 11 SNPs which passed genotyping QC. (PDF) [file pgen.1002158.s005.pdf]

**Supplementary Table 2.** Summary of GWAS and validation results for association with SCD.

| Locus | SNP | Chr | Position | Coded /Non-coded allele | AF | GWAS OR (95% CI) | GWAS P | Validation OR (95% CI) | Validation P | Combined P |
|---|---|---|---|---|---|---|---|---|---|---|
| 1 | rs11590910 | 1 | 91,890,870 | C/G | 0.360 | 0.79 (0.72—0.88) | 9.66E-06 | | | |
| 2 | **rs174230** | **2** | **159,883,556** | **T/C** | **0.013** | **2.49 (1.78—3.47)** | **8.58E-08** | **1.38 (0.99—1.93)** | **0.03** | 2.98E-07 |
| | **rs4665058** | **2** | **159,898,455** | **A/C** | **0.014** | **2.52 (1.80—3.53)** | **7.07E-08** | **1.48 (1.05—2.08)** | **0.01** | ***1.81E-10** |
| 3 | rs16880395 | 4 | 27,848,761 | T/C | 0.230 | 1.30 (1.16—1.46) | 5.14E-06 | 0.97 (0.87—1.07) | 0.27 | 0.01 |
| | rs17619526 | 4 | 27,851,492 | T/A | 0.230 | 1.31 (1.17—1.47) | 4.54E-06 | | | |
| 4 | rs2178490 | 5 | 30,875,088 | G/A | 0.207 | 1.32 (1.17—1.48) | 5.63E-06 | 0.90 (0.81—1.00) | NA | 0.11 |
| 5 | rs1978838 | 5 | 106,008,311 | A/T | 0.234 | 0.76 (0.67—0.85) | 4.19E-06 | | | |
| | rs12517578 | 5 | 106,008,730 | G/C | 0.234 | 0.76 (0.67—0.85) | 4.41E-06 | 0.94 (0.85—1.04) | 0.12 | 0.0001 |
| 6 | rs3193970 | 10 | 97,061,998 | C/T | 0.421 | 0.78 (0.71—0.86) | 1.11E-06 | 0.96 (0.89—1.05) | 0.2 | 0.0001 |
| | rs10748630 | 10 | 97,085,783 | T/C | 0.482 | 1.27 (1.15—1.40) | 1.47E-06 | | | |
| 7 | rs11626637 | 14 | 45,792,412 | G/A | 0.100 | 0.64 (0.52—0.78) | 9.57E-06 | 1 (0.87—1.16) | NA | 0.01 |
| 8 | rs1318021 | 16 | 75,946,589 | A/T | 0.421 | 0.75 (0.68—0.83) | 3.77E-08 | | | |
| | rs2650907 | 16 | 75,950,708 | G/C | 0.421 | 0.75 (0.68—0.83) | 4.39E-08 | 1.03 (0.92—1.13) | NA | 0.0005 |
| 9 | rs9910447 | 17 | 31,496,575 | T/C | 0.117 | 1.92 (1.46—2.51) | 2.43E-06 | | | |
| | rs1024448 | 17 | 31,497,076 | T/C | 0.116 | 1.91 (1.47—2.49) | 1.29E-06 | | | |
| 10 | rs7218928 | 17 | 32,338,069 | G/A | 0.429 | 0.79 (0.72—0.87) | 4.34E-06 | 1.02 (0.94—1.11) | NA | 0.008 |
| 11 | rs12601622 | 17 | 73,560,970 | A/G | 0.014 | 6.79 (3.43—13.42) | 3.69E-08 | 0.89 (0.64—1.23) | NA | 0.08 |
| 12 | rs6507566 | 18 | 39,939,723 | T/G | 0.330 | 1.26 (1.14—1.40) | 7.09E-06 | 0.93 (0.85—1.02) | NA | 0.08 |
| 13 | rs12461046 | 19 | 54,924,441 | A/T | 0.057 | 2.01 (1.49—2.71) | 5.35E-06 | | | |

Chr, chromosome; AF, allele frequency of coded allele (study size weighted average). Follow-up genotyping results are reported for 1,730 SCD cases and 10,530 controls, with the exception of rs12601622 (1,460 SCD cases, 10.182 controls), which failed genotyping in the Oregon-SUDS follow-up study. **Bold** indicates nominal significance ($P$<0.05) for validation. P-values for validation are reported as one-sided, and NA indicates opposite direction of effect from GWAS. *Includes ARREST (719 SCD cases, 4,190 controls) and AGNES (670 SCD cases, 654 controls) studies. Follow-up genotyping results are reported for the 11 SNPs which passed genotyping QC.
