# Supplementary material for: Identification of a Sudden Cardiac Death Susceptibility Locus at 2q24.2 through Genome-Wide Association in European Ancestry Individuals
Source: PLoS Genet. 2011 Jun 30;7(6):e1002158. doi: 10.1371/journal.pgen.1002158 (PMC3128111; doi:10.1371/journal.pgen.1002158)
Supplement: Table S3 — Follow-up genotyping cohort characteristics. Age for prospective studies is at baseline. (PDF) [file pgen.1002158.s006.pdf]

Supplementary Table 3. Follow-up genotyping cohort characteristics.

Characteristic	AGNES	ARREST	CHS	FinGesture	Harvard	CVPI-SCDr	Oregon-SUDS
N, Participants with genotype data	1324	4,909	4429	1440*	1655	259**	771
N, Participants after exclusion	1324	4,909	4429	1071	1655	259	618
Sex, women, %	20.2%	44.8%	56.8%	35.2%	31.4%	14.7%	27.7%
Age, years, mean	56.9	61.2	72.8	57.6	64.3	49.5	61.3
Age, years, range	30-84	15-99	63 - 100	35-94	40-92	35-80	35–80
N, Sudden cardiac death	670	719	199	567	435	259	270
Average time to SCD	NA	NA	6.3	NA	NA	NA	NA
Mean follow up time	NA	NA	10.1	NA	NA	NA	NA
Prospective/Case-control	Case-control	Case-control	Prospective	Case-control	Case-control	Case-control	Case-control
SCD Definition/Ascertainment	In-hospital first acute ST-elevation MI with (case) and without (control) VF	Out of hospital ECG-documented VT/VF, EMS	sudden pulseless condition from a cardiac origin in a previously stable individual, review of death and medical records	out-of-hospital sudden death with evidence of a coronary complication, witnessed within 6 hours of symptoms or seen alive in normal state within 24 hours, autopsy-determined	Cardiac death within 1 hour of onset of symptoms or last seen alive in normal state within 24 hours, death and medical records	Cardiac death within 6 hours of onset of symptoms or last seen alive in normal state within 24 hours, autopsy-determined	Sudden unexpected pulseless condition of likely cardiac origin, if unwitnessed, seen alive and in normal state of health within 24 hours, collected through EMS, review of death and medical records
Control Definition	Non-fatal first acute MI	Population based	NA	Population based	Matched on CAD risk factors	Non-SCD CHS samples (n=4228)	CAD with no history of SCD

Age for prospective studies is at baseline.
