# Supplementary material for: Identification of a Sudden Cardiac Death Susceptibility Locus at 2q24.2 through Genome-Wide Association in European Ancestry Individuals
Source: PLoS Genet. 2011 Jun 30;7(6):e1002158. doi: 10.1371/journal.pgen.1002158 (PMC3128111; doi:10.1371/journal.pgen.1002158)
Supplement: Table S4 — Association of QRS interval associated SNPs with SCD. Chr, chromosome; OR, odds ratio; CI, confidence interval. Trait beta estimates (β) are in milliseconds (ms). P-values are for a two-tailed test. Bold indicates nominal significance (P<0.05). Concordant Effect refers to whether the QRS prolonging allele is associated with increased risk of SCD. QRS interval results are drawn from Sootodehnia et al.14. §This SNPs represent the same genetic effect for QT as rs11970286 in Table S5 (r2 = 0.91). (PDF) [file pgen.1002158.s007.pdf]

Supplementary Table 4. Association of QRS interval associated SNPs with SCD

Nearest Gene	Index SNP	Chr	Position	Coded /Non-coded Allele	Trait β	SCD OR (95% CI)	SCD P	Concordant Effect
C1orf185/RNF11/ CDKN2C/FAF1	rs17391905	1	51,258,161	G/T	-1.35	1.19 (0.85—1.66)	0.30	NO
NFIA	rs9436640	1	61,585,698	G/T	-0.59	0.92 (0.84—1.02)	0.12	YES
CASQ2	rs4074536	1	116,023,009	C/T	-0.42	1.92 (0.82—1.04)	0.14	YES
CRIM1	rs7562790	2	36,585,206	G/T	0.39	1.02 (0.92—1.13)	0.69	YES
HEATR5B/STRN	rs17020136	2	37,159,666	C/T	0.51	0.92 (0.82—1.04)	0.19	NO
SCN5A	rs11708996	3	38,608,927	C/G	0.79	1.03 (0.89—1.18)	0.71	YES
SCN5A	rs11710077	3	38,632,903	T/A	-0.84	0.96 (0.85—1.08)	0.53	YES
SCN10A	rs9851724	3	38,694,939	C/T	-0.66	1.05 (0.95—1.16)	0.35	NO
SCN10A	rs6801957	3	38,742,319	T/C	0.77	0.96 (0.87—1.06)	0.39	NO
**TKT/CACNA1D/ PRKCD**	**rs4687718**	**3**	**53,257,343**	**A/G**	**-0.63**	**1.27 (1.10—1.45)**	**0.0007**	**NO**
LRIG1/SLC25A26	rs2242285	3	66,514,292	A/G	0.37	1.02 (0.92—1.12)	0.72	YES
HAND1/SAP30L	rs13165478	5	153,849,233	A/G	-0.55	0.90 (0.82—1.00)	0.06	YES
CDKN1A	rs9470361	6	36,731,357	A/G	0.87	1.06 (0.94—1.19)	0.38	YES
**C6orf204/SLC35 F1/PLN/ BRD7P3**	**§rs11153730**	**6**	**118,774,215**	**C/T**	**0.59**	**1.13 (1.02—1.25)**	**0.013**	**YES**
TBX20	rs1362212	7	35,078,546	A/G	0.69	1.06 (0.93—1.22)	0.40	YES
IGFBP3	rs7784776	7	46,393,385	G/A	0.39	1.04 (0.94—1.14)	0.48	YES
DKK1	rs1733724	10	53,893,983	A/G	0.49	0.96 (0.84—1.10)	0.57	NO
VTI1A	rs7342028	10	114,469,252	T/G	0.48	1.08 (0.96—1.22)	0.17	YES
TBX5	rs883079	12	113,255,960	C/T	0.49	0.98 (0.87—1.10)	0.69	NO
TBX3	rs10850409	12	113,844,460	A/G	-0.49	0.92 (0.82—1.04)	0.15	YES
KLF12	rs1886512	13	73,418,187	A/T	-0.4	0.95 (0.86—1.05)	0.32	YES
SIPA1L1	rs11848785	14	71,127,108	G/A	-0.5	0.94 (0.84—1.06)	0.32	YES
GOSR2	rs17608766	17	42,368,270	C/T	0.53	1.06 (0.92—1.21)	0.43	YES
PRKCA	rs9912468	17	61,748,819	G/C	0.39	0.99 (0.90—1.09)	0.82	NO
SETBP1	rs991014	18	40,693,884	T/C	0.42	1.01 (0.91—1.11)	0.91	YES

Chr, chromosome; OR, odds ratio; CI, confidence interval. Trait beta estimates (β) are in milliseconds (ms). P-values are for a two-tailed test. **Bold** indicates nominal significance (P<0.05). Concordant Effect refers to whether the QRS prolonging allele is associated with increased risk of SCD. QRS interval results are drawn from Sootodehnia et al.[14]. §This SNPs represent the same genetic effect for QT as rs11970286 in Table 3 (r^2=0.91).
