# Supplementary material for: Identification of a Sudden Cardiac Death Susceptibility Locus at 2q24.2 through Genome-Wide Association in European Ancestry Individuals
Source: PLoS Genet. 2011 Jun 30;7(6):e1002158. doi: 10.1371/journal.pgen.1002158 (PMC3128111; doi:10.1371/journal.pgen.1002158)
Supplement: Table S5 — Association of QT interval associated SNPs with SCD. Chr, chromosome; OR, odds ratio; CI, confidence interval. Trait beta estimates (β) are in milliseconds (ms). P-values are for a two-tailed test. Bold indicates nominal significance (P<0.05). Concordant Effect refers to whether the QT prolonging allele is associated with increased risk of SCD. QT results are drawn from the QTSCD study13, unless otherwise noted. *Genome-wide significant results (P<5×10^-8^) are drawn from the QTGEN study12, and standardized beta estimates and SE were converted to ms using SD = 17.5 ms. §This SNP represent the same genetic effect for QRS interval as rs11153730 in Table S4 (r2 = 0.91). (PDF) [file pgen.1002158.s008.pdf]

Supplementary Table 5. Association of QT interval associated SNPs with SCD

Nearest Gene	Index SNP	Chr	Position	Coded /Non-coded Allele	Trait β	SCD OR (95% CI)	SCD P	Concordant Effect
RNF207	rs846111	1	6,201,957	C/G	1.49	1.03 (0.90—1.18)	0.69	YES
**NOS1AP**	**rs12143842**	**1**	**160,300,514**	**T/C**	**2.88**	**1.16 (1.03—1.3)**	**0.010**	**YES**
NOS1AP	rs4657178	1	160,477,234	T/C	2.19	1.00 (0.89—1.12)	0.95	NO
ATP1B1	rs10919071	1	167,366,107	G/A	-2.05	0.91 (0.78—1.07)	0.24	YES
SCN5A	rs11129795	3	38,568,397	A/G	-1.27	0.96 (0.85—1.08)	0.47	YES
PLN	rs12210810	6	118,759,897	C/G	-3.13	0.84 (0.66—1.06)	0.15	YES
**PLN**	**§rs11970286**	**6**	**118,787,067**	**T/C**	**1.64**	**1.11 (1.01—1.22)**	**0.037**	**YES**
KCNH2	rs2968863	7	150,254,070	T/C	-1.35	0.95 (0.85—1.07)	0.42	YES
KCNH2	rs4725982	7	150,268,796	T/C	1.58*	1.00 (0.89—1.12)	0.98	NO
KCNQ1	rs2074238	11	2,441,379	T/C	-8.22*	0.89 (0.58—1.38)	0.62	YES
**KCNQ1**	**rs12296050**	**11**	**2,445,918**	**T/C**	**1.44**	**0.85 (0.76—0.96)**	**0.014**	**NO**
LITAF	rs8049607	16	11,599,254	T/C	1.25	1.04 (0.93—1.16)	0.48	YES
NDRG4	rs7188697	16	57,179,679	G/A	-1.66	1.04 (0.93—1.17)	0.46	NO
LIG3	rs2074518	17	30,356,290	T/C	-1.23*	0.95 (0.87—1.05)	0.35	YES
KCNJ2	rs17779747	17	66,006,587	T/G	-1.16	0.98 (0.89—1.09)	0.76	YES
KCNE1	rs1805128	21	34,743,550	T/C	4.03*	1.07 (0.77—1.47)	0.70	YES

Chr, chromosome; OR, odds ratio; CI, confidence interval. Trait beta estimates (β) are in milliseconds (ms). P-values are for a two-tailed test. **Bold** indicates nominal significance ($P<0.05$). Concordant Effect refers to whether the QT prolonging allele is associated with increased risk of SCD. QT results are drawn from the QTSCD study[13], unless otherwise noted. *Genome-wide significant results ($P<5x10^{-8}$) are drawn from the QTGEN study[12], and standardized beta estimates and SE were converted to ms using SD=17.5 ms. §This SNP represent the same genetic effect for QRS interval as rs11153730 in Table 2 (r^2=0.91).
