# Supplementary material for: Identification of a Sudden Cardiac Death Susceptibility Locus at 2q24.2 through Genome-Wide Association in European Ancestry Individuals
Source: PLoS Genet. 2011 Jun 30;7(6):e1002158. doi: 10.1371/journal.pgen.1002158 (PMC3128111; doi:10.1371/journal.pgen.1002158)
Supplement: Table S6 — Association of RR interval associated SNPs with SCD. Chr, chromosome; OR, odds ratio; CI, confidence interval. Trait beta estimates (β) are in milliseconds (ms). P-values are for a two-tailed test. Concordant Effect refers to whether the QT prolonging allele is associated with increased risk of SCD. RR results are drawn from11. §This SNP is partially correlated with rs11153730 and rs11970286 from Tables S4 and S5, respectively (r2 = 0.59). (PDF) [file pgen.1002158.s009.pdf]

Supplementary Table 6. Association of RR interval associated SNPs with SCD

Nearest Gene	Index SNP	Chr	Position	Coded /Non-coded Allele	Trait β	SCD OR (95% CI)	SCD P	Concordant Effect
GJA1/HSF2	rs9398652	6	122,187,733	A/C	-12.6	0.87 (0.74–1.02)	0.09	NO
GJA1	rs11154022	6	121,790,241	A/G	5.8	1.09 (0.98–1.22)	0.13	NO
MYH6	rs452036	14	22,935,725	A/G	-7.8	0.94 (0.84–1.05)	0.31	NO
MYH7/NDNG	rs223116	14	23,046,850	A/G	-7.4	1.08 (0.95–1.23)	0.25	YES
SOX5/BCAT1	rs17287293	12	24,662,145	A/G	-8.6	1.09 (0.95–1.26)	0.20	YES
SLC35F1/PLN	§rs281868	6	118,680,754	A/G	6.3	1.09 (0.98–1.2)	0.10	NO
SLC12A9	rs314370	7	100,291,144	T/C	7.6	1.09 (0.96–1.23)	0.18	NO
FADS1	rs174547	11	61,327,359	T/C	6.2	0.99 (0.89–1.1)	0.84	YES
CD34	rs2745967	1	206,195,345	A/G	-5.4	0.98 (0.89–1.09)	0.71	NO

Chr, chromosome; OR, odds ratio; CI, confidence interval. Trait beta estimates (β) are in milliseconds (ms). P-values are for a two-tailed test. Concordant Effect refers to whether the QT prolonging allele is associated with increased risk of SCD. RR results are drawn from[11].
§This SNP is partially correlated with rs11153730 and rs11970286 from Tables 2 and 3, respectively (r^2=0.59).
