# Supplementary material for: Identification of a Sudden Cardiac Death Susceptibility Locus at 2q24.2 through Genome-Wide Association in European Ancestry Individuals
Source: PLoS Genet. 2011 Jun 30;7(6):e1002158. doi: 10.1371/journal.pgen.1002158 (PMC3128111; doi:10.1371/journal.pgen.1002158)
Supplement: Table S7 — Study genome-wide genotyping characteristics. (PDF) [file pgen.1002158.s010.pdf]

Supplementary Table 7. Study genome-wide genotyping characteristics.

Characteristic	ARIC	FHS	FinGesture	Rotterdam	Oregon-SUDS
Array	Affy 6.0	Affy 500K, 50K MIP	Affy 6.0	Illumina 550k	Affy 6.0
Genotype calling software	Birdseed	BRLMM	Birdseed	BeadStudio	Birdseed
SNP call rate exclusion	<95%	<=97%	<97%	<98%	<95%
SNP MAF exclusion	<1%	<1%	<5%	<1%	<1%
pHWE exclusion	<10e-5	<10e-6	<10e-06	<10e-06	<10e-5
Imputation software	Mach1 v1.0.16	Mach 1.0.15	Mach 1.0.15	Mach1 v1.0.15	Mach1 v1.0.16
NCBI Build for imputation	Build 36	Build36	Build36	Build36	Build 36
GWAS statistical analysis	probABEL	R	mach2dat 1.0.10	probABEL	probABEL
Related individuals?	No	Yes	No	No	No
Familial adjustment method	N/A	Kinship package in R	N/A	N/A	N/A
Genomic control factor (λ)	1.001	1.024	1.029	1.012	0.987
